# Lack of Anatomical Concordance between Preablation and Postablation CT Images: A Risk Factor Related to Ablation Site Recurrence

**DOI:** 10.1155/2012/870306

**Published:** 2012-12-24

**Authors:** Petra G. Kele, Eric J. Van der Jagt, Paul F. M. Krabbe, Koert P. de Jong

**Affiliations:** ^1^Department of Radiology, University Medical Center Groningen, University of Groningen, Hanzeplein 1, 9700 RB Groningen, The Netherlands; ^2^Health Technology Assessment Unit, Department of Epidemiology, University Medical Center Groningen, University of Groningen, Groningen, The Netherlands; ^3^Division of Hepato-Pancreatico-Biliary Surgery and Liver Transplantation, Department of Surgery, University Medical Center Groningen, University of Groningen, Groningen, The Netherlands

## Abstract

*Objective*. Variation in the position of the liver between preablation and postablation CT images hampers assessment of treatment of colorectal liver metastasis (CRLM). The aim of this study was to test the hypothesis that discordant preablation and postablation imaging is associated with more ablation site recurrences (ASRs). *Methods*. Patients with CRLM were included. Index-tumor size, location, number, RFA approachs and ablative margins were obtained on CT scans. Preablation and postablation CT images were assigned a “Similarity of Positioning Score” (SiPS). A suitable cutoff was determined. Images were classified as identical (SiPS-id) or nonidentical (SiPS-diff). ASR was identified prospectively on follow-up imaging. *Results*. Forty-seven patients with 97 tumors underwent 64 RFA procedures (39 patients/63 tumors open RFA, 25 patients/34 tumours CT-targeted RFA, 12 patients underwent >1 RFA). Images of 52 (54%) ablation sites were classified as SiPS-id, 45 (46%) as SiPS-diff. Index-tumor size, tumor location and number, concomitant partial hepatectomy, and RFA approach did not influence the SiPS. ASR developed in 11/47 (23%) patients and 20/97 (21%) tumours. ASR occurred less frequently after open RFA than after CT targeted RFA (*P* < 0.001). ASR was associated with larger index-tumour size (18.9 versus 12.8 mm, *P* = 0.011). Cox proportional hazard model confirmed SiPS-diff, index-tumour size >20 mm and CT-targeted RFA as independent risk factors for ASR. *Conclusion*. Variation in anatomical concordance between preablation and postablation images, index-tumor size, and a CT-targeted approach are risk factors for ASR in CRLM.

## 1. Introduction

Liver metastases develop in approximately 50% of patients with colorectal carcinoma. Partial hepatectomy is a potential curative treatment, but only 10–20% of the patients are eligible for partial hepatectomy. Radiofrequency ablation (RFA) is an alternative for patients with unresectable tumours and is often used as an adjunct to partial hepatectomy [1–4]. By using an image-guided approach, electrodes are positioned in the tumour either percutaneously or by an open approach [5–8]. One of the major problems with RFA is incomplete ablation, leading to ablation site recurrences (ASRs) [9]. Factors associated with low ASR rates are small index-tumour size [10], low number of treated tumours [11], at least 10 mm margins of coagulation around the tumour [1, 12], open surgical approach (versus percutaneous CT-targeted approach) [2, 3], and tumor location distant from large vessels [2, 3]. The purpose of post-RFA imaging is the early detection of ASR, providing the opportunity to repeat the RFA procedure. Strategies used for post-RFA evaluation include measuring the ablative margins or focusing on contrast enhancement in the ablation zone. A disadvantage of these techniques is the variation that can occur in the position of the liver between the pre-RFA scan and the post-RFA scan, resulting in an inaccurate quantitative assessment of the ablative margin. This may give false reassurance to the adequacy of the treatment and might thus be an indirect risk factor for development of ASR. In this study, we test the hypothesis that variation in the position of the liver between pre-RFA scan and post-RFA scan makes the assessment of completeness of ablation of colorectal liver metastases difficult, and as an indirect risk factor is associated with the development of future ablation site recurrences (ASRs).

## 2. Patients and Methods

### 2.1. Patients

The study was approved by our institutional review board. Between July 2000 and July 2008, 142 RFA procedures were performed for primary (benign and malignant) and secondary liver tumours in our center. Sixty-five percent of these procedures were done with an open approach, 35% was performed percutaneously under CT targeting. Open procedures were performed in patients who also underwent a partial hepatectomy or if the tumour could not be safely reached using the percutaneous route. Laparoscopic procedures were not performed. All procedures were performed by one of the authors, an experienced hepatobiliary surgeon in collaboration with dedicated radiologists. CT-targeted RFA was performed in collaboration with a radiologist. Fifty-two patients (37%) underwent RFA for colorectal liver metastases. Five patients were excluded—missing pre-RFA images (*n* = 2), multiple and widespread liver metastases shortly after RFA making assessment of ASR impossible (*n* = 2), and lost to followup (*n* = 1). Thus, 47 patients who underwent 64 RFA procedures for 97 liver metastases were included in the study. Partial hepatectomy was performed as described previously and is considered the gold standard [13]. It is standard praxis to fix the liver remnant after partial hepatectomy with the aim to keep the liver remnant in the same position in order to prevent rotation of the remaining liver lobe. Rotation can lead to torsion of the draining hepatic vein and congestion of the liver lobe. RFA was only performed if partial hepatectomy was not able to render the liver tumor-free.

### 2.2. RFA Procedure

RFA was performed by one staff HPB surgeon (K. P. de Jang) in collaboration with a staff radiologist (E. J. Van der Jagt) for the CT-guided procedures. Ablation procedures are performed in our hospital since 1995 with about 20 procedures per year for colorectal liver metastases. We used the RF 3000 TM Radio Frequency Ablation System (Boston Scientific, Boston, MA, USA). A LeVeen electrode of 2, 3.5, 4, or 5 cm diameter was used, depending on tumour diameter. The RFA electrode was positioned using ultrasonography in open and CT-guided in CT-targeted RFA. RFA was applied according to the protocol of the manufacturer. RFA was continued until the generation of radiofrequency waves was blocked by the rise in tissue impedance. Large tumours were treated by several overlapping positions of the deployed RFA electrode. Terminology used in this paper is in accordance to the guidelines given by Goldberg et al. [14].

### 2.3. CT Protocol

Patients underwent triphasic CT scanning before the RFA procedure, one week after the RFA procedure, then at three-monthly intervals during the first two years and every six months thereafter. CT was performed on a 16- or 64-slice multidetector CT scanner (S OMATOM Sensation 64, Siemens, Erlangen, Germany). Intravenous contrast was used, 120 mL iodixanol 320 mg I/mL (Visipaque 320, GE Healthcare, Chalfont St Giles, UK), with a flow rate of 4.0 mL/sec. All subjects were scanned in craniocaudal direction during inspiratory breath-holding. CT images were acquired in a supine position using a 16 × 1.5 (16-slice) or 24 × 1.2 (64-slice) collimation, tube potential 120 kV, tube current time product 130 mAs, pitch 1, slice thickness of 2 mm, reconstruction Kernel B30f, and reconstruction increment 1.5.

### 2.4. Followup

Followup of the ablated tumours consisted of CT imaging or F18-fluorodeoxyglucose positron emission tomography (FDG-PET) when CT imaging was inconclusive. Patients were considered to have recurrences when there was a typical pattern of contrast enhancement on CT imaging and/or pathological glucose uptake on PET scanning. 

### 2.5. Post-RFA Evaluation

**Figure 1:**
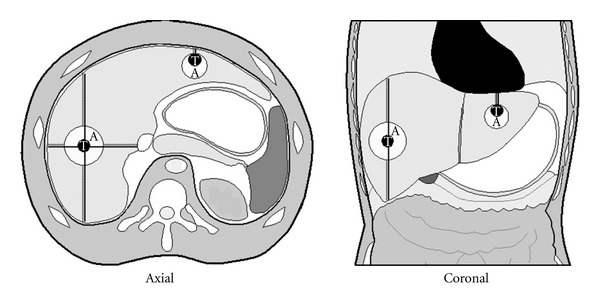
Explanation of the method of measurement. Schematic representation of the method of measurement of the ablation zone. Figure 1 representing the axial and coronal view respectively of the tumour (black circle with white T) and the ablation zone (white circle with black A). Ablative margins were calculated as follows. The distance from the edge of the tumour to the surface of the liver was measured in all six directions on the pre-RFA scan (continuous line). The same measurements were performed for the post-RFA scan from the edge of the ablation zone scan to the surface of the liver (dotted line). The ablative margins are the difference between both distances. The tumour in the left liver lobe is considered to be incompletely ablated because in one of the six directions the difference between both distances is zero.

**Figure 2:**
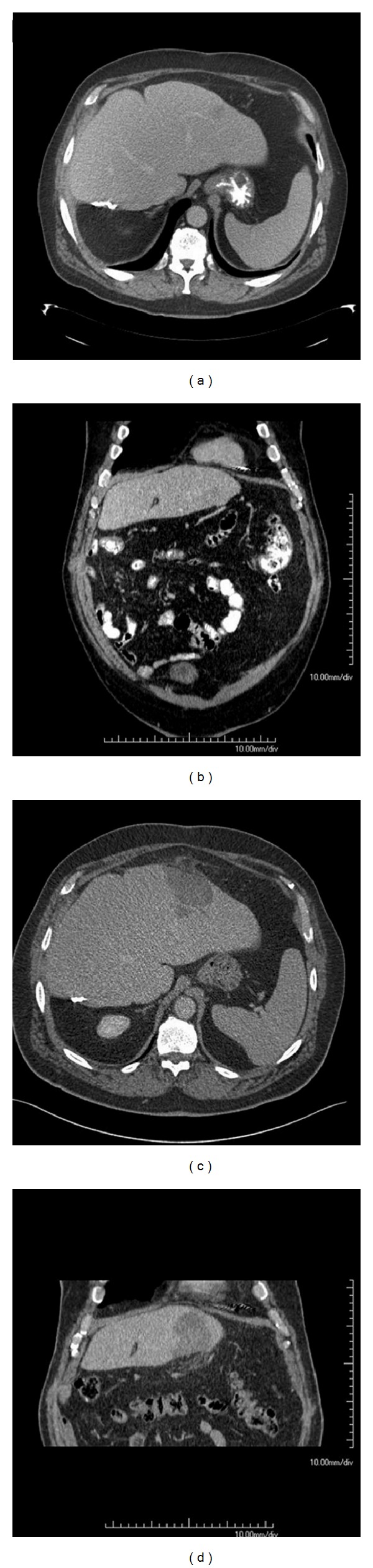
Similarity of Positioning Score-identical (SiPS-id). Example of identical (concordant) pre-RFA CT images and post-RFA CT images, classified as Similarity of Positioning Score-identical (SiPS-id). The pre-RFA CT scan ((a) axial, (b) coronal) and the post-RFA CT scan ((c) axial, (d) coronal) are well comparable.

**Figure 3:**
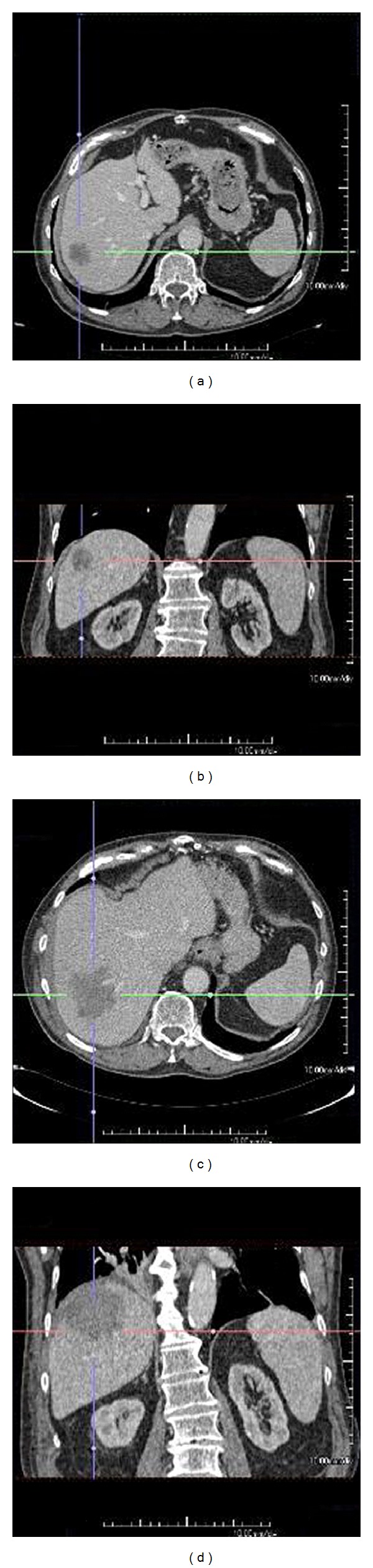
Similarity of Positioning Score-different (SiPS-diff). Example of nonidentical (discordant) pre-RFA CT-images and post-RFA CT-images, classified as Similarity of Positioning Score-different (SiPS-diff). The pre-RFA CT-scan ((a) axial, (b) coronal) and post-RFA CT-scan ((c) axial, (d) coronal) are not comparable.

Radiological evaluation of the tumours before and one week after the RFA procedure was performed on an Aquarius Workstation (version 1.8.3.6, TeraRecon Inc., San Mateo, CA, USA). Images in axial and reconstructed coronal planes were used for three-dimensional measurements and comparison of the pre-RFA scan and the post-RFA scan (explanation in Figure 1). This was done by two of the authors (P. G. Kele, E. J. Van der Jagt). Reliable comparison was only possible when the position of the liver was identical or almost identical on the pre-RFA scan and the post-RFA scan. Therefore, a dichotomous “Similarity of Positioning Score” (SiPS) was developed in which post-RFA scans were compared to pre-RFA scans. Post-RFA scans were centrally and blindly classified as SiPS-identical (SiPS-id, i.e., comparable to the pre-RFA scans, Figure 2) or SiPS-different (SiPS-diff, i.e., not comparable to the pre-RFA scan, Figure 3). A post-RFA scan was considered SiPS-id when the vascular configuration (especially hepatic and portal veins) was identical or nearly identical to that on the pre-RFA scan. In addition, the projection of the abdominal organs, bony structures (vertebrae and ribs), and the position of previously placed surgical clips had to be identical or nearly identical. When these criteria were not met, a scan was regarded as SiPS-diff. For validation of SiPS, one of the authors (P. G. Kele) classified all tumours twice for intra-observer agreement. Another radiologist with two years CT experience performed the same classifications to obtain the interobserver agreement. Ablative margins >10 mm were considered sufficient. The smallest margin in one of the six directions was considered the most imperfect one. Therefore, tumours with an ablative margin <10 mm in only one of the six directions were regarded as having an insufficient ablative margin.

### 2.6. Definition of Ablation Site Recurrence

**Figure 4:**
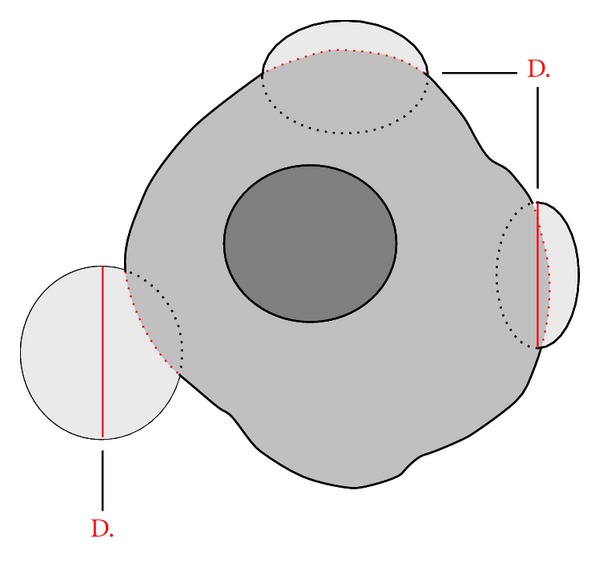
Definition of ablation site recurrence. Examples of ablation site recurrences (ASRs). The largest diameter of both lesions on the right is in direct contact with the ablation zone. The lesion on the left is not an ASR, because the center of the line representing the largest diameter is not in direct contact with the ablation zone. It is more probably a satellite metastasis which was already present at the time of the ablation. Outgrowth of this lesion took place after the RFA procedure. This prerequisite is necessary to prevent erroneously identified outgrowing satellite lesions in the close vicinity of the ablated tumour as ASR.

Progression at the site of a previously RFA-treated tumour was considered ASR when it met both of the following criteria: (1) growth of a contrast-enhancing lesion within or directly adjacent to the ablation zone and (2) the largest diameter of the lesion was in direct contact with the ablation zone. The latter prerequisite is to exclude the outgrowth of satellite lesions in the vicinity of the ablated tumour (Figure 4).

### 2.7. Statistical Analysis

Chi-square and Fisher's exact test were applied to assess the relationship between categorical variables SiPS (identical versus different), RFA approach (open versus CT-targeted), partial hepatectomy in the history (yes versus no), number of tumours ablated (<3 versus ≥3), localization of tumours (subcapsular, i.e., <10 mm under the liver capsule, versus central), ablative margins (>10 mm versus <10 mm and >5 mm versus <5 mm), and ASR (yes versus no). For comparing the continuous variable index-tumour size between tumours with and without ASR, Student's *t*-test was used after correction for nonnormal distribution (log transformation). Survival was assessed with Kaplan-Meier analysis. Variables possibly contributing to ASR were analyzed by using log-rank test. Cox proportional hazard model was used to identify independent risk factors for ASR. Kappa statistics were calculated to test the intraobserver and interobserver agreement of SiPS. Agreement was rated as poor (kappa 0–0.2), fair (kappa 0.21–0.40), moderate (kappa 0.41–0.60), substantial (kappa 0.61–0.80), or excellent (kappa 0.81–1.0) [15]. The significance level was set at a *P* < 0.05 for all tests. Statistical analysis was performed using SPSS (Statistical Package for Social Sciences version 16.0 Inc., Chicago, IL, USA). 

## 3. Results

### 3.1. General Characteristics and Recurrence Patterns

In 47 patients with 97 colorectal liver metastases, 64 RFA procedures were performed. An open approach was used in 39 patients with 63 metastases. Percutaneous CT-targeted RFA was performed in 25 patients with 34 metastases. There were 12 patients who underwent one or more further RFA procedures, of which 5 patients had repeat RFA for ASR. ASR was seen in 11 patients (23%) with 20 metastases (21%). There were 2 patients (4%) with 3 metastases (3%) who showed ASR without recurrences elsewhere. Recurrent disease elsewhere occurred in 33 patients (70%) with 74 ablated liver metastases (76%) and was concomitant with ASR in 9 patients (19%) with 17 metastases (18%). Recurrence without ASR was seen in 24 patients (51%) with 57 tumours (59%) (Table 1). Mean index-tumour size before RFA was 13.9 mm (SD 1.8, range 3.9–78.0 mm).

### 3.2. Similarity of Positioning Score (SiPS)

After CT-targeted RFA, 15 of the 34 tumours (44%) were classified as SiPS-id, the remaining 56% as SiPS-diff. After open RFA, 37 of the 63 tumours (59%) were classified as SiPS-id, the remaining 41% as SiPS-diff. After open RFA with concomitant partial hepatectomy, 24 tumours were classified as SiPS-id (56%), the remaining 44% as SiPS-diff. Kappa statistics for intraobserver agreement were excellent (kappa 0.834, *P* < 0.001) and substantial for interobserver agreement (kappa 0.752, *P* < 0.001). Index-tumour size, RFA approach, concomitant partial hepatectomy, number of ablated tumours, and tumour localization were not different in the SiPS-diff group versus the SiPS-id group (Table 2).

### 3.3. ASR

ASR occurred in 20 of 97 metastases (21%). Tumours with ASR were larger than tumours without ASR (18.9 mm versus 12.8 mm, *P* = 0.011). ASR was seen in 17 (50%) tumours treated with CT-targeted RFA and 3 (5%) tumours with open RFA (*P* < 0.001). ASR was seen in 6 (12%) tumours classified as SiPS-id and 14 (31%) tumours classified as SiPS-diff (*P* = 0.017). ASR was not different in tumours with ablative margins <5 mm (*P* = 0.464).

Univariate analysis showed more ASR in tumours treated with CT-targeted RFA (*P* < 0.001), in tumours classified as SiPS-diff (*P* = 0.023), and in tumours with an index-tumour size >20 mm (*P* = 0.009). Tumour localization (subcapsular versus central) and ablative margins were not associated with ASR (*P* = 0.483 and *P* = 0.576, resp.). Cox proportional hazard model identified RFA approach, SiPS, and index-tumour size as independent predictors of ASR. CT-targeted RFA was associated with the highest risk for developing ASR, followed by SiPS-diff and an index-tumor size >20 mm (Table 3). 

### 3.4. Survival

Median time of followup was 36 months (interquartile range 25–49). Median overall survival in the open RFA group was 40.7 months (95%-CI 23.3–58.2) and was not statistically different for the CT-targeted RFA group (*P* = 0.23). As the proportion of disease-free patients in the latter group was more than 50% at the end of the study, the median survival could not be estimated. After open RFA, median disease-free survival was 35.2 months (95%-CI 29.7–40.7) and 32.6 months (95%-CI 15.8–49.5) after CT-targeted RFA (*P* = 0.50).

## 4. Discussion

RFA is increasingly used in patients with malignant liver tumors in whom partial hepatectomy is not able to render the liver tumor-free. RFA seems to be a highly attractive treatment modality since it is associated with lower morbidity and mortality compared to partial hepatectomy. However, a major concern is the reported high incidence of ablation site recurrences (ASRs). Early evaluation of the completeness of RFA—followed by immediate repeated RFA in case of an incomplete procedure—is essential to reduce the high incidence of ASR. A prerequisite for evaluation of the completeness of RFA is the anatomical concordance or comparability of the pre-RFA scan with the post-RFA scan. In the present study, we hypothesized that incomparability of the pre-RFA scan and the post-RFA scan may result in an increased number of future ASR, since completeness of ablation cannot be evaluated reliably. Indeed we found that this incomparability is a risk factor associated with ASR. Other risk factors were CT-targeted RFA approach (as opposed to open RFA) and an index-tumour size >20 mm.

The reason for using the Similarity of Positioning Score (SiPS) in this study was to evaluate the problem and consequences of incomparable pre-RFA imaging and post-RFA imaging. Fifty-four percent of the post-RFA scans were classified as anatomically concordant or SiPS-identical (SiPS-id), the remaining 46% as anatomically discordant or SiPS-different (SiPS-diff). Open RFA and CT-targeted RFA were equally represented, suggesting that SiPS is not influenced by RFA approach and concomitant partial hepatectomy. Although intuitively it seems reasonable to expect that partial hepatectomy is associated with a change in position and configuration of the liver and thus influence SiPS, we did not encounter this. A probable explanation is that the liver remnant is fixed in position at the end of the operation. This means that SiPS is determined by other factors, for example, changes in the position of the liver as a result of longitudinal or rotational movements of the liver related to variations in diaphragm position. These factors could result in substantial organ position differences. These issues are well known in the field of radiotherapy and nuclear medicine. In radiotherapy, this problem is improved by using implanted markers which optimize accurate tumour targeting and advanced scanning techniques such as four-dimensional CT planning [16, 17]. In nuclear medicine, movements—particularly respiratory movements—can result in mismatch between PET and CT images. Respiratory-motion tracking systems, mathematical correction models, or scanning correction models and post-processional motion-correction methods are used to minimize this problem [18, 19]. These techniques could be useful in reducing organ position differences between subsequent scans in the post-RFA followup.

Radiological evaluation of RFA procedures can be performed by different strategies. Firstly, ablative margins can be estimated by fusing pre-RFA images and post-RFA images. Unfortunately, this method is often hindered by incomparable pre-RFA images and post-RFA images. Secondly, comparing surfaces, volumes, or diameters of the index tumor and post-RFA ablation zone is often not reliable because of geometrical constraints or incomplete overlap of the index tumor and ablation zone [7, 20]. Thirdly, evaluation can be performed by focusing on post-RFA contrast enhancement, which might be misleading because of contrast enhancement associated with post-RFA inflammation and contrast enhancement due to residual tumour origin. Differentiation between these entities can be performed by their different morphological characteristics and contrast-enhancement patterns on multiphase CT scanning [21, 22], but remains difficult.

A possible solution to detect residual tumour after RFA without being hindered by incomparable pre-RFA images and post-RFA images is to perform PET-CT. PET is reported to have a high diagnostic accuracy in detecting residual tumour after RFA compared to contrast-enhanced CT and even MRI, modalities which are more readily available [21, 23]. Until now, only few studies with small patient populations assessed the usefulness of PET-CT after RFA. Based on our study it might be that PET-CT is the preferred imaging modality to detect incomplete ablations in patients with discordant pre-RFA scans and post-RFA scans. A potential limitation might be a false-positive result because of glucose uptake associated with post-RFA inflammation in the early post-RFA period [24]. Another possibility to evaluate the completeness of the ablation is to monitor ablation zone volume on consecutive CT scans, since an ongoing decline in ablation zone volume on consecutive scans is highly predictive of complete ablation and that an increase in volume is associated with ASR [25]. However, this is only noticed later in the followup and not on the first postprocedural scan. Therefore, we recommend that in case of SiPS-id, patients undergo regular followup with multiphase CT scanning every three months in the first two years after the RFA procedure and biannually thereafter. Patients with SiPS-diff should be followed in a similar fashion, but it might be advisable in these patients to perform additional PET-CT scanning three to six months after the RFA procedure, when the post-RFA inflammation has subsided and eventual glucose uptake can be attributed to the residual tumour.

We report an ASR rate of 23% per patient and 21% on a tumour basis. Previously reported ASR rates vary widely between 1.8 and 55% [2, 3, 11, 26–28]. We found more ASR in tumours with SiPS-diff classified scans, CT-targeted RFA treated tumours and tumours with a diameter of >20 mm. Although some studies have shown a higher incidence of ASR with ablation margins <10 mm [29–31], our findings are in line with that of others who have reported that ASR is not related to ablative margins [31, 32]. Unfortunately, authors often do not mention their evaluation methods, which may lead to contradictory reports because of the use of different techniques.

It has been reported that CT-targeted RFA is associated with a higher risk of ASR [2, 3, 12, 26, 28, 33–35], which is in accordance with our results. The most important explanation for the higher ASR rate in CT-targeted RFA is limited access to the tumour compared to open RFA, leading to inadequate ablation. Open RFA allows complete mobilization of the liver, better electrode accessibility, additional manoeuvres (Pringle), and tumour visibility (using intraoperative ultrasound). Especially relevant is our finding that despite the higher incidence of ASR in CT-targeted RFA, survival is not different from patients treated with open RFA. This can very likely be explained by thorough postprocedural followup. By carefully monitoring patients, early detection of ASR offers the possibility of timely interventions such as repeat RFA or partial hepatectomy.

This study has certain limitations. Firstly, the newly introduced SiPS classification system is used in a small patient population. We are planning to validate the SiPS classification in a larger patient cohort. Secondly, we did not perform biopsies to confirm the diagnosis of ASR. However, the reason not to do so is well founded—biopsies can be associated with tumour seeding. This risk outweighs the benefit of the procedure [36, 37]. Although SiPS was described in a relative small population encompassing 97 tumours, it is highly reproducible as reflected by the excellent intraobserver and substantial interobserver agreement.

In conclusion, lack of anatomical concordance between pre-RFA images and post-RFA images, CT-targeted RFA, and index-tumour size >20 mm are independent risk factors associated with future ablation site recurrences. Anatomical concordance of pre-RFA images and post-RFA images, expressed in the Similarity of Positioning Score, is important in evaluating the RFA procedure. In discordant scans, no reliable judgement can be made about the completeness of ablation and thus whether an additional RFA is necessary. Therefore, it is associated with an indirect increased risk of future ablation site recurrences.

## Figures and Tables

**Table 1:** Patient and tumour characteristics.

	Patients	Tumours
Number	47	97
Gender ♂/♀	30/17 (64%/36%)	—
Age (mean, range)	61.8 years (39–81)	—
Deceased	12/47 (26%)	—
Partial hepatectomy	34 (72%)	73 (75%)
Before RFA^a^	5 (15%)	20 (27%)
During RFA	25 (73%)	43 (59%)
After RFA	4 (12%)	10 (14%)
Type of partial hepatectomy (*n* = 34)		
Right-sided hemihepatectomy	12 (35%)	—
Left-sided hemihepatectomy	8 (24%)	—
Segment 2 and 3 resection	12 (35%)	—
Other	2 (6%)	—
Synchronous/metachronous disease	26/21 (55%/45%)	57/40 (59%/41%)
Indication RFA		
Bilobar disease	29 (62%)	—
Recurrence after partial hepatectomy	8 (17%)	—
Major comorbidity	7 (15%)	—
Minimal residual disease	2 (4%)	—
Severe steatosis	1 (2%)	—
RFA procedures		
1 RFA	35 (75%)	—
2 RFAs	9 (19%)	—
3 RFAs	1 (2%)	—
4 RFAs	2 (4%)	—
RFA approach (64 procedures)		
Open	39 (61%)	63 (65%)
CT targeted^b^	25 (39%)	34 (35%)
No. of tumors ablated (64 procedures)		
1 tumor	44 (69%)	—
2 tumors	13 (20%)	—
≥3 tumors	7 (11%)	—
Recurrence	33 (70%)	74 (76%)
ASR^c^	11 (23%)	20 (21%)
Repeat RFA for ASR		
Yes	5 (11%)	10 (10%)^d^
No	42 (89%)	87 (90%)
Partial hepatectomy for ASR		
Yes	1 (2%)	2 (2%)
No	46 (98%)	95 (98%)

^a^RFA: radiofrequency ablation.

^b^CT targeted: computer tomography targeted.

^c^ASR: ablation site recurrence.

^d^CT targeted RFA was performed initially in all tumours which underwent repeated RFA for ASR.

**Table 2:** Effect of different factors on the Similarity of Positioning Score (SiPS).

	SiPS-id^a^ (*n* = 52)	SiPS-diff^a^ (*n* = 45)	*P* value
Index-tumor size (mean ± SD)^b^	14.1 mm (1.9)	13.6 mm (1.8)	0.773
RFA approach^c^			
Open RFA (*n* = 63)	37 (59%)	26 (41%)	0.203
CT-targeted RFA (*n* = 34)	15 (44%)	19 (56%)	
Partial hepatectomy during RFA^c^			
Yes (*n* = 43)	24 (56%)	19 (44%)	0.838
No (*n* = 54)	28 (52%)	26 (48%)	
No. of tumours ablated^c^			
1-2 (*n* = 70)	40 (57%)	30 (43%)	0.364
≥3 (*n* = 27)	12 (44%)	15 (56%)	
Localization^c^			
Subcapsular (*n* = 76)	44 (58 %)	32 (42 %)	0.140
Central (*n* = 21)	8 (38 %)	13 (62 %)	

^a^SiPS: Similarity of Positioning Score, identical (SiPS-id) or different (SiPS-diff).

^b^Student's *t*-test.

^c^Chi-square or Fisher's exact test.

**Table 3:** Cox proportional hazard model showing the relative risk for development of ablation site recurrence compared to the reference standard (1.0).

	Relative risk (95% CI)	*P *value
RFA approach		
Open RFA	1.0	0.001
CT-targeted RFA	9.5 (2.6–34.0)	
Similarity of Positioning Score (SiPS)		
SiPS-identical	1.0	0.019
SiPS-different	3.9 (1.2–12.3)	
Index-tumour size		
<20 mm	1.0	0.010
≥20 mm	3.6 (1.4–9.4)	
